# Radiological features do not predict failure of two-stage arthroplasty for prosthetic joint infection: a retrospective case–control study

**DOI:** 10.1186/1471-2474-15-300

**Published:** 2014-09-10

**Authors:** Susanna Dunachie, James Teh, Vivian Ejindu, Philip Bejon, Hemant Pandit, Ivor Byren

**Affiliations:** Bone Infection Unit, Nuffield Orthopaedic Centre, Oxford University Hospitals NHS Trust, Oxford, UK; Mahidol-Oxford Tropical Medicine Research Unit, Bangkok, Thailand; Department of Radiology, Nuffield Orthopaedic Centre, Oxford University Hospitals NHS Trust, Oxford, UK; Department of Orthopaedic Surgery, Nuffield Orthopaedic Centre, Oxford University Hospitals NHS Trust, Oxford, UK

**Keywords:** Joint infection prosthesis, Outcome measures, Radiology

## Abstract

**Background:**

The management of prosthetic joint infection is complex and there is a lack of standardisation of approaches. We evaluated the role of plain film radiography in predicting prosthesis failure after the first stage of a two-stage revision procedure in a retrospective case–control study.

**Methods:**

Plain films for 41 patients aged 46 to 87 years (mean 69) were assessed by two musculoskeletal specialist radiologists for seven features (retained or new metalwork, retained cement or restrictor, new fracture, local antimicrobial delivery system and drain) we hypothesised may predict for failure. Inter-observer agreement was assessed by Kappa score and logistic regression analysis was performed to evaluate the relationship of the seven radiological features adjusting for patient age, gender and number of previous revisions.

**Results:**

There was substantial inter-observer agreement, with a Kappa score of 0.73 (95% CI 0.72-0.74) for all data points collected. Concordance was 100% for evaluating the presence or absence of an antimicrobial delivery system or drain, with lower consensus for evaluating cement (Kappa 0.60, 95% CI 0.35-0.84) and fractures (Kappa 0.59, 95% CI 0.31-0.87). None of the variables’ conditions significantly predicted failure.

**Conclusions:**

Our findings support the opinion that surgical expertise which maximizes removal of foreign material is sufficient in conjunction with antibiotic therapy.

**Electronic supplementary material:**

The online version of this article (doi:10.1186/1471-2474-15-300) contains supplementary material, which is available to authorized users.

## Background

Prosthetic joint infection (PJI) is a miserable complication of surgery that was undertaken to improve quality of life, and management requires skilled decision making. PJI occurs in up to 2% of primary hip and knee arthroplasties[1, 2] although reporting rates vary. Successful management of PJI requires eradication of infection and restoration of joint function. The “gold standard” management for complex cases has been advocated as two-stage revision[3, 4], consisting of removal of as much prosthetic material and cement as possible, followed by a prosthesis-free period with adjuvant intravenous/oral antibiotic therapy before a new prosthesis is sited, although excellent outcomes from single stage revisions are achievable in selected patients with favourable characteristics[5]. In two-stage revision plain film X-ray imaging post removal of the infected prosthesis is current practice, but there are limited data on its role in predicting for failure after re-implantation. Our unit, a national referral centre for prosthetic joint infection, provided an ideal setting for such a study, using a registry of patient characteristics and outcomes[6]. We therefore conducted a retrospective case–control study of prosthetic joint infection cases managed by two-stage revision at our unit, to evaluate the role of plain film imaging post first-stage.

## Methods

### Study population

Cases and controls with at least 6 months of follow-up data were identified from the registry of patients undergoing two-stage revision surgery for prosthetic joint infection at our national referral centre. There was no research-related contact with patients. In response to our enquiry, our institutional review board (Oxfordshire Research Ethics Committee) advised informed consent and ethical approval was not required. All activity was conducted in accordance with the Declaration of Helsinki, and national and institutional standards. Prosthetic joint infection was defined as patients having a clinical syndrome of arthroplasty infection (any of persistent inflammation in the tissues around the implant, wound discharge or implant loosening) with one or more of the following: bacterial growth of an indistinguishable organism from two or more deep periprosthetic tissue samples; histology of periprosthetic tissues indicative of infection; or a persistent sinus tract.

**Figure 1:**
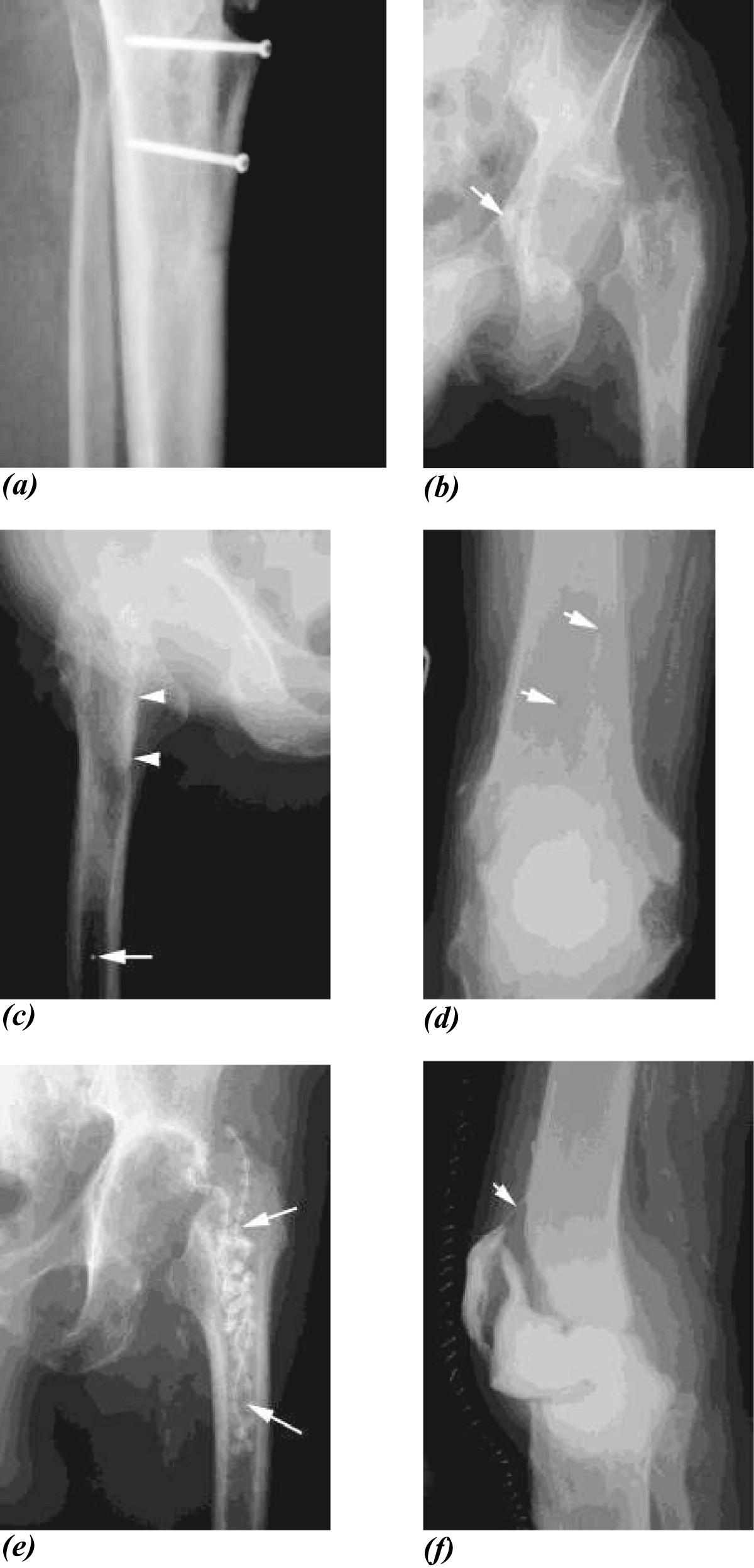
**Post first-stage radiographs were examined for the presence or absence of the following seven features: retained metalwork (not shown), new metalwork (Figure** **1** **a), retained cement (Figure** **1** **b), retained restrictor (a radio-opaque plug inserted in the medullary canal during prosthesis placement to restrict the area of cement, Figure** **1** **c), new fracture (Figure** **1** **d), local antimicrobial delivery system (for example gentamicin loaded beads, Figure** **1** **e) and drain (Figure** **1** **f).**

Patients were managed by a specialist multi-disciplinary team including orthopaedic surgeons and infectious diseases physicians. All patients underwent intra-operative sampling in multiple of infected material for microbiology and histology at the first stage (removal of infected prosthesis). Antibiotic therapy was as described previously[6]. Briefly, patients received empirical intravenous meropenem and vancomycin post-operatively and the antibiotic therapy was rationalized once microbiological culture results were available. Patients received six weeks of intravenous therapy as the gold standard and had a minimum of two weeks without antibiotics prior to second stage. Despite most patients having a minimum of two weeks without antibiotics prior to first-stage surgery, culture-negative results were common in this cohort[6]. Coagulase negative staphylococci were the micro-organisms most frequently isolated, followed by *Staphylococcus aureus* and diptheroids. We analysed the post first-stage radiographs for seven described and novel criteria that we hypothesized may predict for failure. The presence or absence of the following seven features was assessed: retained metalwork (not shown), new metalwork (Figure1a), retained cement (Figure1b), retained restrictor (a radio-opaque plug inserted in the medullary canal during prosthesis placement to restrict the area of cement, Figure1c), new fracture (Figure1d), local antimicrobial delivery system (for example gentamicin loaded beads, Figure1e) and drain (Figure1f). Treatment failure was defined as sinus drainage after reimplantation, a requirement for revision surgery or amputation.

### Radiograph review

Subjects in this study underwent surgery before the hospital installed PACS (Picture archiving and communication systems) for viewing radiographs electronically. Therefore radiograph films were obtained from an off-site archive. Radiographs were reviewed independently by two specialist musculoskeletal radiologists blinded to outcome. Pre-operative radiographs were used to aid analysis, where available.

### Analysis

Statistical analysis was performed using *SPSS/PAWS Statistics 18* and *Stata 10*. Inter-observer consensus was evaluated by Kappa score. Binary logistic regression analysis for the seven variables was performed with correction for age, gender, and number of previous revisions, and odds ratios calculated with 95% confidence intervals. Significance was judged to be a *P* value of less than 0.05. Results were expressed graphically with *GraphPad Prism 5*.

## Results and discussion

### Subject characteristics

66 subjects were identified from our database of which 41 had imaging available to allow inclusion in the study. Patients had undergone the first stage of revision surgery between January 1999 and June 2004 and follow-up was continued until July 2011 or until the endpoint of treatment failure was reached. The range of follow-up was from 8 months to 11 years and 6 months (mean 7 years 2 months). For the 25 subjects for whom no films were available, this was because the radiograph packet covering the period of surgery was not found in the archive for 19 subjects and because no post first-stage radiograph had been performed in the remaining 6 cases. The post first-stage radiograph was performed on average six days after the first-stage operation. Pre-operative films were available for comparison in 33 out of 41 cases. There were 12 cases and 29 controls. Male gender was associated with failure (*P* = 0.028). The number of previous revisions ranged from 0 to 4, with increasing number of revisions associated with the outcome of failure in the logistic regression model (*P* = 0.02). The subject characteristics are shown in Table 1. There were no statistically significant differences between cases and controls for age and site.

**Table 1:** **Subject characteristics**

*Condition*		*Number cases*	*Number controls*
		*N = 12*	*N = 29*
‘Age	mean years	66.9	70.4
	(range)	(46–78)	(56–87)
Gender	Male	10	14
	Female	2	15
Joint	Hip	4	17
	Knee	8	12
Number of previous revisions	0 previous	5	20
	1	5	4
	2	1	3
	3	0	0
	4	1	0
	unknown	0	2

The characteristics of the cases and controls the study are displayed.

### Inter-observer consensus

**Figure 2:**
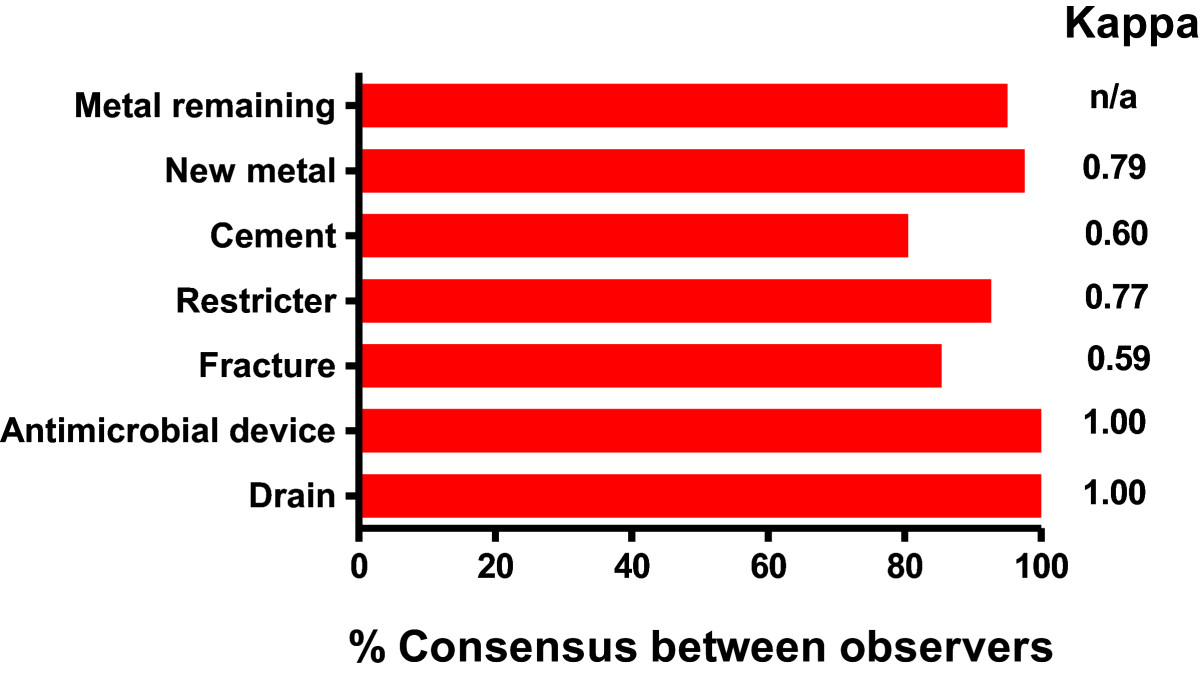
**Two musculoskeletal specialist radiologists assessed plain radiographs post first-stage revision for the presence or absence of seven features.** The inter-observer consensus of the two radiologists is shown.

The presence or absence of the seven conditions was scored independently by the two radiologists. There was substantial inter-observer agreement, with a Kappa score of 0.73 (95% CI 0.72-0.74) for all data points collected. Concordance was 100% for evaluating the presence or absence of an antimicrobial delivery system or drain, with lower consensus for evaluating cement (Kappa 0.60, 95% CI 0.35-0.84) and fractures (Kappa 0.59, 95% CI 0.31-0.87) [Figure2].

### Analysis for prediction of failure

**Figure 3:**
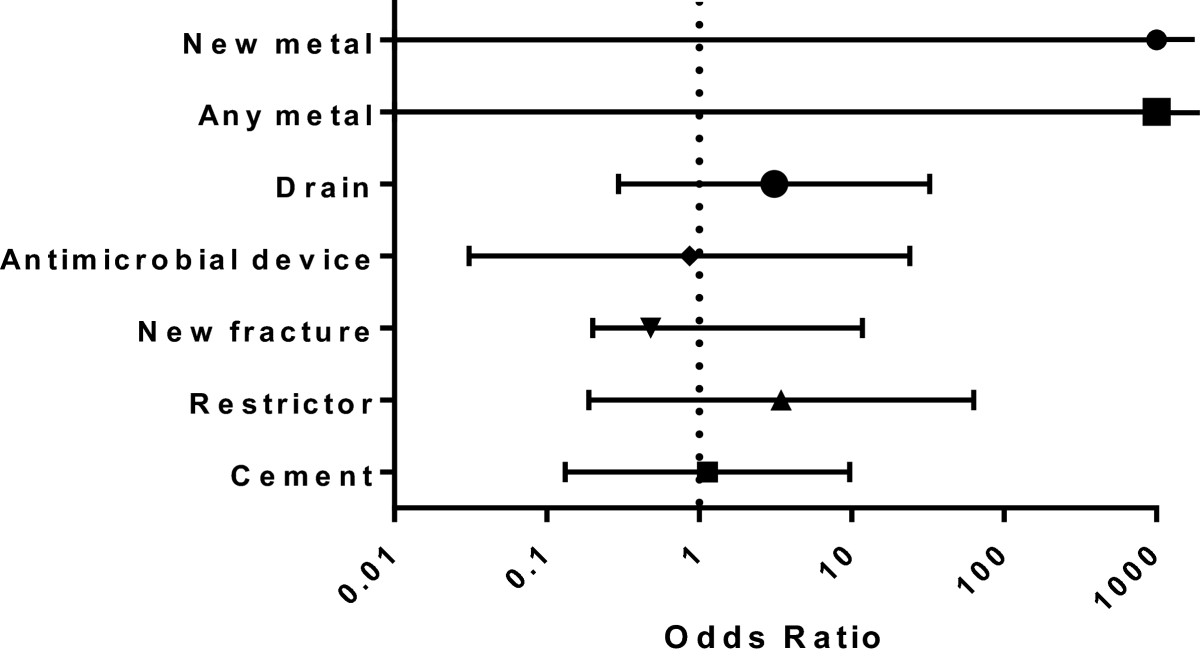
**The odds ratios for the presence of each condition compared to the absence predicting prosthesis failure are shown.**

Logistic regression analysis was performed for the seven conditions adjusting for patient age and gender and number of previous revisions, with odds ratios calculated [Table2, Figure3]. None of the variables assessed significantly predicted failure.

**Table 2:** **Analysis for prediction of failure (binary logistic regression)**

*Condition*	*Number cases*	*Number controls*	*P value*	*Odds ratio(OR)*	*95% C.I. for OR*	
	*N = 12*	*N = 29*			Lower	Upper
Metal remaining	1 (8.3%)	0 (0%)	1.0	1 x 10^10^	0	∞
New metal	1 (8.3%)	0 (0%)	1.0	1 x 10^10^	0	∞
Cement	4 (33.3%)	9 (31%)	0.909	1.134	0.132	9.739
Restrictor	2 (16.7%)	4 (13.8%)	0.404	3.449	0.189	63.108
New fracture	3 (25%)	5 (17.2%)	0.654	0.481	0.020	11.807
Antimicrobial device	1 (8.3%)	3 (10.3%)	0.932	0.865	0.031	24.047
Drain	3 (25%)	4 (13.8%)	0.345	3.108	0.296	32.623

The table displays binary logistic regression analysis performed for seven variables with the outcome of prosthesis failure, with the model adjusted for age, gender and number of previous revisions.

### Discussion

We report a case–control study of 41 subjects on the predictive value of plain film imaging after first-stage revision of infected prosthesis. The study did not find an association between any of the seven features examined and outcome of implant failure. In particular, the study did not demonstrate any correlation between increased risk of failure i.e. reinfection and presence of retained metalwork or retained cement or retained cement restrictor. This is contrary to some previously published reports. McDonald *et al*. reported on the results of 82 hips which were treated with two-stage revision for infected hip replacement[7]. In particular the authors report on the significantly increased risk of recurrent sepsis in patients with retained cement at the time of first-stage revision. The authors also suggest that the second stage revision surgery i.e. re-implantation should preferably be postponed for at least one year after the first-stage revision. Tsukayama *et al*. reported on 106 infected hip replacements[8] and concluded that the factors associated with recurrent infection were retained bone cement, the number of previous operations, potential immunocompromise, and early postoperative infection after arthroplasty without cement. Although complete removal of all foreign material and postponing the second intervention as long as possible have been the gold standard, both these studies were conducted more than 20 years ago and since then significant improvements have been made in the diagnosis, medical treatment and surgical interventions for infected joint replacements. Indeed a recently published series of 15 infected hip replacement cases managed with two-stage revision also reported excellent outcomes despite retention of the original well-fixed femoral cement mantle[9]. Ability to identify the appropriate organisms as well as treat them adequately with correct antibiotics has improved. Instead of removing all the retained foreign material, the tendency by surgeons now is to remove the material which is loose and/or easily accessible. This is particularly relevant to retained cement as the distinction between retained cement and bony cortex can at times be very difficult if not impossible. If the interface between retained cement and bone is solid i.e. cement is not obviously loose then one need not remove this cement as invariably this will lead to increased risk of fracture and prolonged surgical time. Both these risks are associated with prolonged surgical time and delayed recovery. The difficulty in identifying retained cement radiologically is reflected in this variable having one of the lowest Kappa score in this study for inter-observer agreement (0.60). It is possible that computed tomography imaging (CT) could identify retained cement more reliably but this is not current practice.

As none of the seven factors studied correlated with a risk of increased failure, one may question the need for obtaining plain radiographs after the first-stage revision surgery. Although the radiographs may not predict success or failure of first-stage revision surgery i.e. need for further debridement for recurrent infection, they still have a crucial role to play. The radiographs confirm the placement of cement spacer (if used) and these baseline radiographs can be compared with subsequent radiographs to ensure that the spacer has not moved out of position and/or there is no bony erosion. In addition, the radiographs can demonstrate presence of a fracture which can inadvertently occur in a porotic bone and this has clinical implications about the weight bearing status after the first-stage revision surgery. Although the presence of a retained restrictor may not increase the risk of infection, their existence should be noted by the treating surgeon as these retained restrictors may prevent use of a longer implant during the second stage revision.

The relatively high consensus of opinion for two musculoskeletal radiologists reviewing the radiographs for seven features is encouraging although it is possible that this could not be achieved by non-specialist radiologists.

There are certain limitations of this study. This study has small numbers due to the relatively low rate of failure following two-stage revision for PJI and therefore was not powered to detect small effects. However we are able to conclude that for the management of prosthetic joint infection there is no increased risk of failure if retained foreign material is noted on the plain radiographs post first-stage revision surgery. The impact of type of organism on development of recurrent sepsis has previously been explored in this population and was not found to be significant[6].

This study was performed in a university teaching hospital which provides expertise in multi-disciplinary fields including a dedicated bone infection unit. Our unit gets tertiary referrals from all over the country and has set algorithms for managing prosthetic joint infection. The surgeons, the bone infection unit physicians, microbiologists and radiologists work very closely and this helps in identifying and following the optimal treatment options for managing these demanding cases.

## Conclusions

This study provides novel data for an under-researched field and suggests that plain-film radiological evidence of retained foreign material in our unit does not predict for failure in two-stage revision arthroplasty. Good consensus of opinion was found between two musculoskeletal radiologists in assessing radiographs. Although plain radiographs after first-stage revision for infection cannot predict failure, their use in clinical practice is imperative as they help the surgeon decide the post-operative management with particular reference to the mobilization and extent of weight bearing that can be safely allowed.

## Authors’ original submitted files for images

Below are the links to the authors’ original submitted files for images. Authors’ original file for figure 1 Authors’ original file for figure 2 Authors’ original file for figure 3

## Abbreviations

PJI: prosthetic joint infection.
