# Supplementary figures and images for: Radiological features do not predict failure of two-stage arthroplasty for prosthetic joint infection: a retrospective case–control study
Source: BMC Musculoskelet Disord. 2014 Sep 10;15:300. doi: 10.1186/1471-2474-15-300 (PMC4168065; doi:10.1186/1471-2474-15-300)

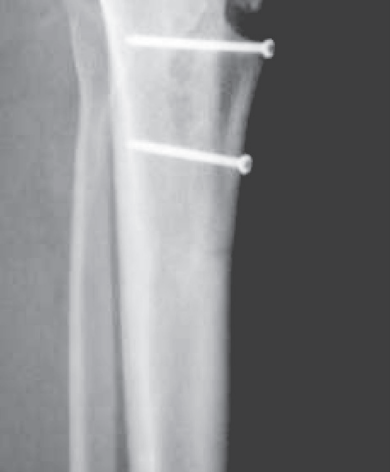

*(a)*

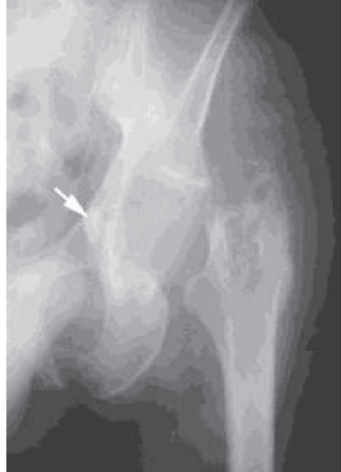

*(b)*

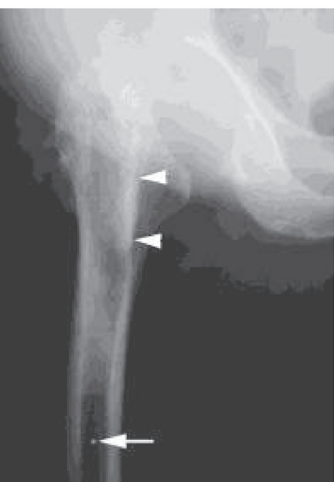

*(c)*

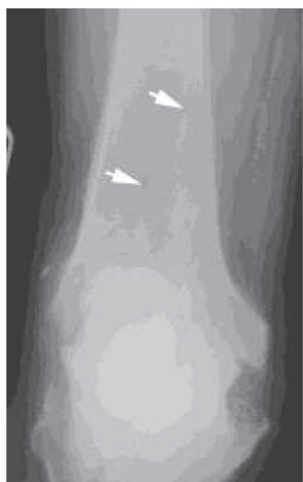

*(d)*

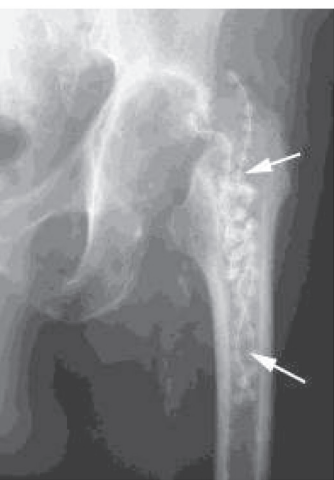

*(e)*

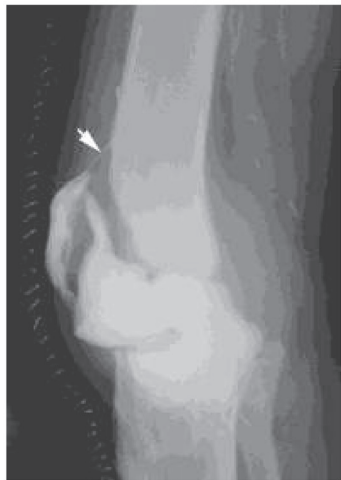

*(f)*

Supplement: Supplementary file 1 — Authors’ original file for figure 1 [file 12891_2013_2242_MOESM1_ESM.pdf]

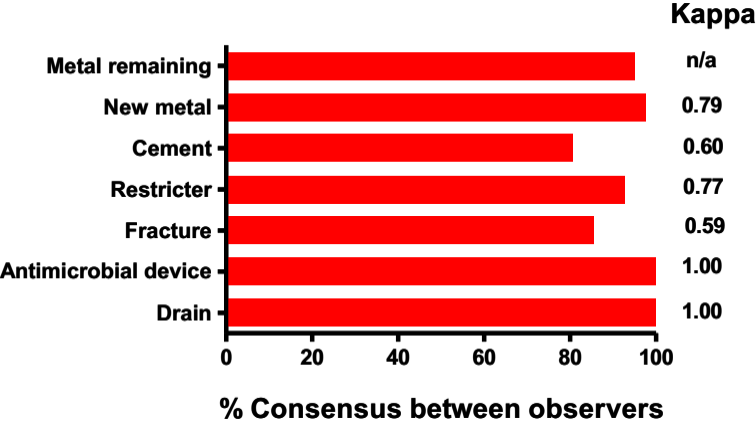

Supplement: Supplementary file 2 — Authors’ original file for figure 2 [file 12891_2013_2242_MOESM2_ESM.pdf]

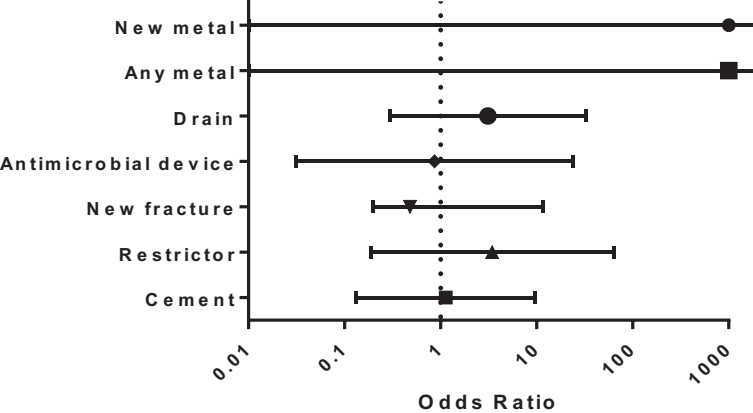

Supplement: Supplementary file 3 — Authors’ original file for figure 3 [file 12891_2013_2242_MOESM3_ESM.pdf]
